# Calculating the power of a planned individual participant data meta‐analysis of randomised trials to examine a treatment‐covariate interaction with a time‐to‐event outcome

**DOI:** 10.1002/jrsm.1650

**Published:** 2023-06-29

**Authors:** Richard D. Riley, Gary S. Collins, Miriam Hattle, Rebecca Whittle, Joie Ensor

**Affiliations:** ^1^ Institute of Applied Health Research, College of Medical and Dental Sciences University of Birmingham Birmingham UK; ^2^ Centre for Statistics in Medicine, Nuffield Department of Orthopaedics, Rheumatology and Musculoskeletal Sciences University of Oxford Oxford UK; ^3^ School of Medicine Keele University Keele UK

**Keywords:** individual participant data (IPD) meta‐analysis, power, sample size, treatment effect modifiers, treatment‐covariate interactions

## Abstract

Before embarking on an individual participant data meta‐analysis (IPDMA) project, researchers should consider the power of their planned IPDMA conditional on the studies promising their IPD and their characteristics. Such power estimates help inform whether the IPDMA project is worth the time and funding investment, before IPD are collected. Here, we suggest how to estimate the power of a planned IPDMA of randomised trials aiming to examine treatment‐covariate interactions at the participant‐level (i.e., treatment effect modifiers). We focus on a time‐to‐event (survival) outcome with a binary or continuous covariate, and propose an approximate analytic power calculation that conditions on the actual characteristics of trials, for example, in terms of sample sizes and covariate distributions. The proposed method has five steps: (i) extracting the following aggregate data for each group in each trial—the number of participants and events, the mean and SD for each continuous covariate, and the proportion of participants in each category for each binary covariate; (ii) specifying a minimally important interaction size; (iii) deriving an approximate estimate of Fisher's information matrix for each trial and the corresponding variance of the interaction estimate per trial, based on assuming an exponential survival distribution; (iv) deriving the estimated variance of the summary interaction estimate from the planned IPDMA, under a common‐effect assumption, and (v) calculating the power of the IPDMA based on a two‐sided Wald test. Stata and R code are provided and a real example provided for illustration. Further evaluation in real examples and simulations is needed.

**Highlights**

**What is already known?**

- Individual participant data (IPD) meta‐analysis projects potentially allow more robust and powerful examinations of participant‐level relationships, such as whether participant‐level covariates interact with treatment effect (treatment‐covariate interactions).
- However, IPD projects are time‐consuming and so, before IPD collection, their potential power should be examined, to help inform whether they are worth investment.

**What is new?**

- We derive an approximate analytic approach for calculating the power of a planned IPD meta‐analysis to estimate treatment‐covariate interactions using IPD from multiple randomised trials with a time‐to‐event outcome.
- Our approach uses (published) trial aggregate data, to condition the power calculation on the actual characteristics of trials promising IPD, for example, in terms of sample sizes and covariate distributions, assuming exponential survival distributions.

**Potential impact for RSM readers outside the authors' field?**

- The approach can be applied to any IPD meta‐analysis project aiming to model interactions with time‐to‐event (survival) data.

## 1 INTRODUCTION

Individual participant data (IPD) meta‐analysis projects obtain, check, harmonise and meta‐analyse the IPD from multiple studies to address a particular research question.^1^ They are considerable undertakings, often taking upwards of 2 years to complete, and so should not be embarked upon without due thought. This should include determining how many trials are likely to provide their IPD and, based on this, estimating the potential power of the planned IPD meta‐analysis.^2^, ^3^, ^4^ Such power estimates are needed to help inform whether the IPD meta‐analysis project is worth the time and funding investment, before IPD are collected. For example, it is potentially unwise to spend 2 years collecting IPD if the power of a subsequent IPD meta‐analysis is potentially only 20%; conversely, if the power is likely to be over 80% then this gives a strong rationale for time and resource investment.

We have previously described how to calculate the power when planning an IPD meta‐analysis of randomised trials with either a continuous or binary outcome,^3^, ^5^ where the estimand of interest is a treatment‐covariate interaction at the participant level. Participant‐level covariates that interact with treatment effect are also known as effect modifiers, predictive markers (especially in the oncology literature), and subgroup effects. The premise is that the magnitude of treatment effect is conditional on the value of the participant‐level covariate. An example is the interaction between the effect of trastuzumab and a breast cancer patient's oestrogen receptor status. Single trials are typically powered on the overall treatment effect, and so rarely have the power to detect genuine treatment‐covariate interactions, and this provides one of the key motivations for combining IPD from multiple trials in an IPD meta‐analysis.

In this article we propose a new method to calculate the power of a planned IPD meta‐analysis project to estimate a treatment‐covariate interaction for a time‐to‐event (survival) outcome. Many IPD meta‐analysis projects involve survival outcomes, for example, in cancer and cardiovascular trials where long‐term follow‐up and outcomes are of interest. Further, compared to our earlier work for continuous and binary outcomes,^3^, ^5^, ^6^ the extension to survival outcomes is complicated by the issue of censored observations and the need to account for length of follow‐up. This is problematic in advance of IPD collection, but here we propose approximate closed‐form (analytic) approaches to power calculations that only require readily available aggregate data to be obtained per trial.

The paper outline is as follows. In Section 2 we describe an analytic solution for Fisher's information matrix and the variance matrix of parameter estimates from an exponential survival regression model of a single randomised trial, and apply them to a real example with comparison of results to those from a Cox model. The analytic solutions are used within Section 3, as part of a four‐step process to calculate the approximate power of a planned IPD meta‐analysis project to estimate a treatment‐covariate interaction for a survival outcome, in advance of IPD collection. The four steps are: (1) extract aggregate data from trial publications; (2) derive variances of treatment‐covariate interactions for each trial separately, conditional on the aggregate data extracted and assumptions about the magnitude of the treatment‐covariate interaction and an exponential survival distribution; (3) calculate the variance of the summary treatment‐covariate interaction from the planned IPD meta‐analysis, under a common‐effect assumption, which is a function of the trial‐specific variances from the previous step; and (4) use the result to calculate the corresponding power of the planned IPD meta‐analysis project based on a two‐sided Wald test. Stata and R code are provided to implement the approach. Section 4 illustrates the methodology with two examples, and Section 5 concludes with discussion, noting that further evaluation in real examples and simulations is needed.

## 2 ESTIMATING THE VARIANCE OF A TREATMENT‐COVARIATE INTERACTION FROM AN EXPONENTIAL SURVIVAL REGRESSION MODEL FOR A SINGLE TRIAL

In this section, we focus on the analysis of a *single* randomised trial to examine a treatment‐covariate interaction, and the statistical theory for obtaining the variance of a treatment‐covariate interaction estimate. This will be used in Section 3 within our proposed power calculation for IPD meta‐analysis projects.

Let i denote a particular trial in the IPD meta‐analysis and j denote a participant in that trial. We focus on a parallel‐group trial design, comparing a treatment ($x_{\mathrm{ij}}=1$) to a control ($x_{\mathrm{ij}}=0$). Let $z_{\mathrm{ij}}$ be a participant‐level covariate (e.g., the age of participant j in trial i), observed for all participants in each trial, and $t_{\mathrm{ij}}$ denotes the event time for participant j. The estimand of interest is the interaction between the treatment effect (as measured by a log hazard ratio) and the covariate $z_{\mathrm{ij}}$. In this article, we will model this using an exponential regression framework, under a proportional hazards assumption, as follows.

### 2.1 Exponential regression model specification

The exponential regression model with a hazard rate of $\eta_{\mathrm{ij}}$ for participant j in trial i can be written as:

(1) $$t_{\mathrm{ij}}\sim \text{exponential}\left(\eta_{\mathrm{ij}}\right)\;\;\;\ln\left(\eta_{\mathrm{ij}}\right)=\mu_{\mathrm{ij}}=\alpha_{i}+\beta_{i}x_{\mathrm{ij}}+\gamma_{i}z_{\mathrm{ij}}+\lambda_{i}x_{\mathrm{ij}}z_{\mathrm{ij}}$$

As each trial is analysed separately the trial subscript i is not strictly required, but we retain it as the solutions that follow will be used in subsequent sections to derive power calculations for the IPD meta‐analysis setting, for which the i notation is needed. Apart from the intercept, all parameters correspond to log hazard ratios and these are assumed constant over time. The treatment‐covariate interaction is denoted by $\lambda_{i}$, and is adjusted for the prognostic effect ($\gamma_{i}$) of the covariate of interest ($z_{\mathrm{ij}})$ and the reference treatment effect ($\beta_{i})$. Other prognostic factors could also be adjusted for, but we do not consider this here. The intercept, $\alpha_{i}$, denotes the baseline hazard (rate).

Our focus is on estimating the treatment‐covariate interaction term, $\lambda_{i}$, which indicates the expected change in treatment effect (log hazard ratio) for a one‐unit increase in $z_{\mathrm{ij}}$ for trial i. For a continuous covariate, this assumes the effect of the interaction is linear. Although extension to non‐linear trends is important in practice,^5^ for simplicity assuming a linear relationship will be sensible for the power calculation that follows in Section 3.

The exponential regression model of Equation (1) can equivalently be written as an accelerated failure time model, as follows,^7^

(2) $$\ln\left(t_{\mathrm{ij}}\right)=-\mu_{\mathrm{ij}}+\varepsilon_{\mathrm{ij}}=-\alpha_{i}-\beta_{i}x_{\mathrm{ij}}-\gamma_{i}z_{\mathrm{ij}}-\lambda_{i}x_{\mathrm{ij}}z_{\mathrm{ij}}+\varepsilon_{\mathrm{ij}}$$

where $\varepsilon_{\mathrm{ij}}$ follows an extreme value distribution with pdf $f\left(\varepsilon_{\mathrm{ij}}\right)=\exp\left(\varepsilon_{\mathrm{ij}}\right)\exp\left(-\exp\left(\varepsilon_{\mathrm{ij}}\right)\right)$. We will use this specification when deriving the variance matrix of parameter estimates, but the interpretation of parameters is identical to before for the exponential regression (in particular, $\lambda_{i}$ is the treatment‐covariate interaction).

### 2.2 Deriving Fisher's information matrix and the variance matrix of parameter estimates

Using the IPD from a particular trial, the parameters in Equation (1) or (2) can be estimated using maximum likelihood estimation, for example, using an iterative approach such as Newton–Raphson. The variance of parameter estimates can then be calculated using the inverse of Fisher's information matrix. Of key interest for our power calculation is deriving an analytic expression for the estimated variance of ${\hat{\lambda }}_{i}$ for a particular trial. A complication is the need to account for censored observations. Let $y_{\mathrm{ij}}$ be the minimum of log event time and log censoring time for participant j in trial i, and the design matrix $X={\left(1,x_{\mathrm{ij}},z_{\mathrm{ij}},x_{\mathrm{ij}}z_{\mathrm{ij}}\right)}^{\prime }$. The 4 by 4 observed information matrix ($I_{i})$ after fitting Equation (2) for a particular trial can be expressed as^7^, ^8^:

(3) $$\begin{array}{c} I_{i}=XX^{\prime }\exp\left(y_{\mathrm{ij}}+{\hat{\mu }}_{\mathrm{ij}}\right)\\ I_{i}=\left[\begin{array}{cccc} \sum_{i=1}^{n_{i}}w_{\mathrm{ij}} & \sum_{i=1}^{n_{i}}x_{\mathrm{ij}}w_{\mathrm{ij}} & \sum_{i=1}^{n_{i}}z_{\mathrm{ij}}w_{\mathrm{ij}} & \sum_{i=1}^{n_{i}}x_{\mathrm{ij}}z_{\mathrm{ij}}w_{\mathrm{ij}}\\ \sum_{i=1}^{n_{i}}x_{\mathrm{ij}}w_{\mathrm{ij}} & \sum_{i=1}^{n_{i}}x_{\mathrm{ij}}^{2}w_{\mathrm{ij}} & \sum_{i=1}^{n_{i}}x_{\mathrm{ij}}z_{\mathrm{ij}}w_{\mathrm{ij}} & \sum_{i=1}^{n_{i}}x_{\mathrm{ij}}^{2}z_{\mathrm{ij}}w_{\mathrm{ij}}\\ \sum_{i=1}^{n_{i}}z_{\mathrm{ij}}w_{\mathrm{ij}} & \sum_{i=1}^{n_{i}}x_{\mathrm{ij}}z_{\mathrm{ij}}w_{\mathrm{ij}} & \sum_{i=1}^{n_{i}}z_{\mathrm{ij}}^{2}w_{\mathrm{ij}} & \sum_{i=1}^{n_{i}}x_{\mathrm{ij}}z_{\mathrm{ij}}^{2}w_{\mathrm{ij}}\\ \sum_{i=1}^{n_{i}}x_{\mathrm{ij}}z_{\mathrm{ij}}w_{\mathrm{ij}} & \sum_{i=1}^{n_{i}}x_{\mathrm{ij}}^{2}z_{\mathrm{ij}}w_{\mathrm{ij}} & \sum_{i=1}^{n_{i}}x_{\mathrm{ij}}z_{\mathrm{ij}}^{2}w_{\mathrm{ij}} & \sum_{i=1}^{n_{i}}x_{\mathrm{ij}}^{2}z_{\mathrm{ij}}^{2}w_{\mathrm{ij}} \end{array}\right] \end{array}$$

where $n_{i}$ is the total sample size of trial i, $w_{\mathrm{ij}}=\exp\left(y_{\mathrm{ij}}+{\hat{\mu }}_{\mathrm{ij}}\right)$, ${\hat{\mu }}_{\mathrm{ij}}$ is an individual's estimated linear predictor value from the fitted exponential regression (i.e., ${\hat{\mu }}_{\mathrm{ij}}={\hat{\alpha }}_{i}+{\hat{\beta }}_{i}x_{\mathrm{ij}}+{\hat{\gamma }}_{i}z_{\mathrm{ij}}+{\hat{\lambda }}_{i}x_{\mathrm{ij}}z_{\mathrm{ij}}$) and $XX^{\prime }$ is a 4 by 4 matrix due to the four parameters in the regression equation ($\alpha_{i}$, $\beta_{i}$, $\gamma_{i}$, $\lambda_{i}$). The diagonal elements of the inverse of $I_{i}$ provide the corresponding variances of the four parameter estimates, and of particular interest is the variance of the interaction estimate,

(4) $$\mathrm{var}\left({\hat{\lambda }}_{i}\right)=I_{i}^{-1}\left(4,4\right)$$

where $I_{i}^{-1}\left(4,4\right)$ denotes the 4,4 element of the inverse of the observed information matrix (I) for trial i.

Note that we can decompose the information matrix for a trial into the product of the total sample size and a matrix of expected values,

(5) $$\begin{array}{c} I_{i}=n_{i}\;E_{\left(x,z,w\right)}\left[\begin{array}{cccc} w_{\mathrm{ij}} & x_{\mathrm{ij}}w_{\mathrm{ij}} & z_{\mathrm{ij}}w_{\mathrm{ij}} & x_{\mathrm{ij}}z_{\mathrm{ij}}w_{\mathrm{ij}}\\ x_{\mathrm{ij}}w_{\mathrm{ij}} & x_{\mathrm{ij}}^{2}w_{\mathrm{ij}} & x_{\mathrm{ij}}z_{\mathrm{ij}}w_{\mathrm{ij}} & x_{\mathrm{ij}}^{2}z_{\mathrm{ij}}w_{\mathrm{ij}}\\ z_{\mathrm{ij}}w_{\mathrm{ij}} & x_{\mathrm{ij}}z_{\mathrm{ij}}w_{\mathrm{ij}} & z_{\mathrm{ij}}^{2}w_{\mathrm{ij}} & x_{\mathrm{ij}}z_{\mathrm{ij}}^{2}w_{\mathrm{ij}}\\ x_{\mathrm{ij}}z_{\mathrm{ij}}w_{\mathrm{ij}} & x_{\mathrm{ij}}^{2}z_{\mathrm{ij}}w_{\mathrm{ij}} & x_{\mathrm{ij}}z_{\mathrm{ij}}^{2}w_{\mathrm{ij}} & x_{\mathrm{ij}}^{2}z_{\mathrm{ij}}^{2}w_{\mathrm{ij}} \end{array}\right]\\ =E_{\left(x,z,w\right)}\left[B\right]\\ =n_{i}\;I_{i}^{*} \end{array}$$

where $E_{\left(x,z,w\right)}\left[B\right]$ denotes the expected value of B over the joint distribution of $x_{\mathrm{ij}}$, $z_{\mathrm{ij}}$ and $w_{\mathrm{ij}}$, and $I_{i}^{*}$ is the subsequent 4 by 4 matrix of expected values (also known as the unit information matrix). This decomposition will be helpful in the next section, and allows us to derive the variance of ${\hat{\lambda }}_{i}$ using:

(6) $$\mathrm{var}\left({\hat{\lambda }}_{i}\right)=I_{i}^{*-1}\;\left(4,4\right)/n_{i}$$

### 2.3 Estimates from Cox regression compared to exponential regression

In practice, trial analyses typically use a Cox regression model, which makes no assumption about the shape of the baseline hazard. However, in this article we utilise an exponential regression framework, which assumes a constant baseline hazard over time. This is a pragmatic decision as it allows us to use the analytic solution for Fisher's information matrix as derived in Equations (5) and (6), which (see Section 3) we need to approximate based on only trial aggregate data (e.g., as available from trial publications) in advance of IPD collection. Such an analytic approach is more challenging with a Cox regression model.

**FIGURE 1:**
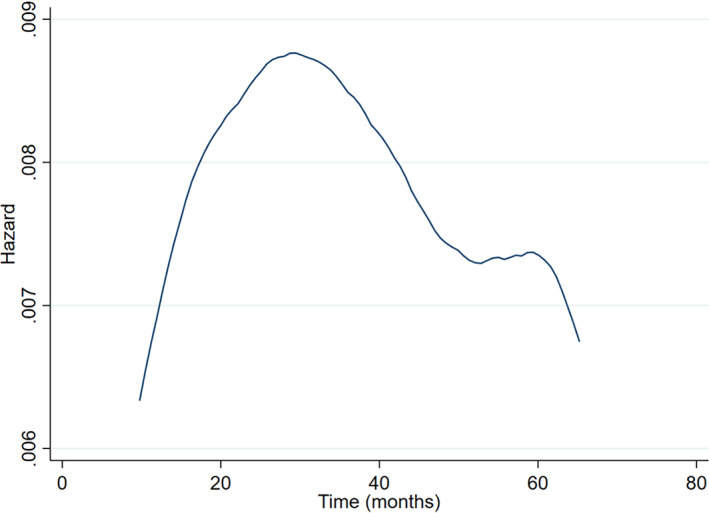
Baseline hazard following a Cox regression model including treatment, age and their interaction, as estimated for the prostate cancer trial described in Section 2.3 using a kernel smoother. [Colour figure can be viewed at wileyonlinelibrary.com]

**FIGURE 2:**
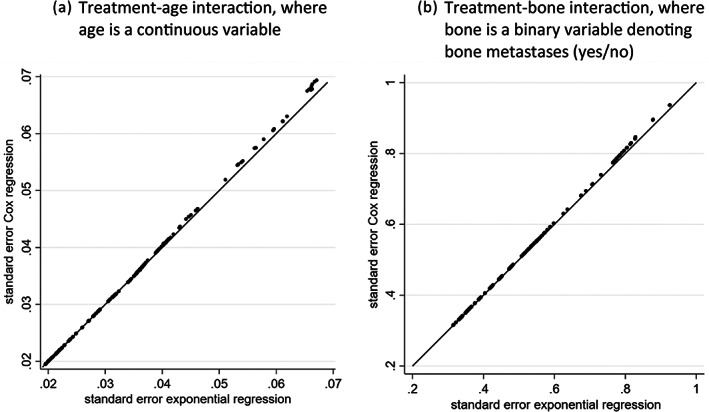
Comparison of Cox regression and exponential regression SEs of the (a) treatment‐age interaction and (b) treatment‐bone interaction from the prostate cancer trial for subsets of participants for increasing sample sizes from 50 participants (far right) to 502 participants (far left).

Reassuringly, parameter estimates and SEs in Cox and exponential regression models will often be similar, even when the hazard rate is not a constant. To illustrate this, consider re‐analysis of IPD from a randomised trial evaluating the use of oestrogen for the treatment of Stage 3 or 4 prostate cancer patients,^9^, ^10^ obtained from http://hbiostat.org/data courtesy of the Vanderbilt University Department of Biostatistics. The trial contains 502 participants and 338 deaths, with a mean follow‐up of 36 months. There are 127 and 375 participants in the placebo and oestrogen groups, respectively. The overall unadjusted treatment effect and SE from a Cox regression model are the same as those from an exponential regression model (log hazard ratio = −0.115, SE = 0.123). Similarly, the treatment‐age interaction and SE are almost identical for the Cox regression (interaction = 0.0166, SE = 0.0198) and exponential regression (interaction = 0.0165, SE = 0.0198). This is despite the observed baseline hazard not being a constant (Figure 1). In small sample sizes differences are more likely to arise. For example, we compared the SE of the treatment‐age interaction in randomly selected subsets of the trial data from a sample size of 50 participants to a full sample size of 502 participants (Figure 2a). Differences are more noticeable in the smaller datasets, with the exponential model producing slightly smaller SEs, but the differences are negligible. For example, with 50 participants, the SEs are 0.070 and 0.067 for the Cox and exponential models, respectively. The same picture is observed when looking at the interaction between treatment and bone metastases (Figure 2b). This gives reassurance that our pragmatic focus on the exponential model is sensible for our power calculation approach that follows.

### 2.4 SEs based on an approximation of Fisher's information matrix

Our power calculations in the next section aim to calculate, for a particular trial, Fisher's information matrix (using Equation 5) and the subsequent variance matrix of parameter estimates (using Equation 6) based on only a trial's published aggregate data. A major difficulty with this is that Fisher's information depends on the value of $w_{\mathrm{ij}}=\exp\left(y_{\mathrm{ij}}+{\hat{\mu }}_{\mathrm{ij}}\right)$ for each participant, and thus depends on $\exp\left(y_{\mathrm{ij}}\right)$ (i.e., the exponential of the minimum of log event time and log censoring time), which will not be available without IPD. To address this, we consider the following three options, which gradually increase in complexity in terms of the aggregate data required.

**FIGURE 3:**
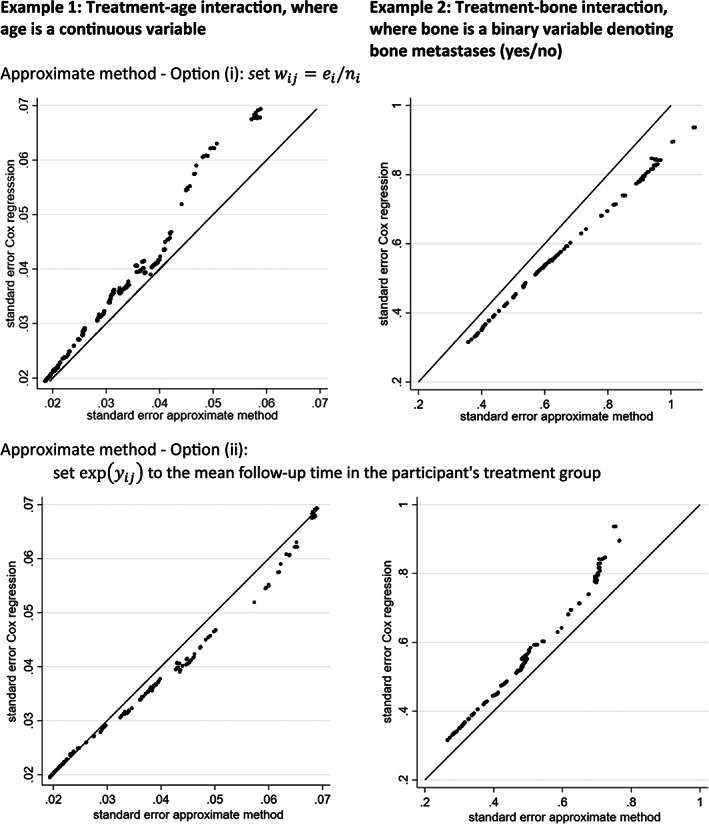
Comparison of SE of a treatment‐covariate interaction as calculated from a Cox regression and from an approximate method based on exponential regression, for a prostate cancer trial for increasing sample sizes from 50 participants (far right points on each graph) to 502 participants (far left points on each graph). Two approximate methods are considered.

**FIGURE 4:**
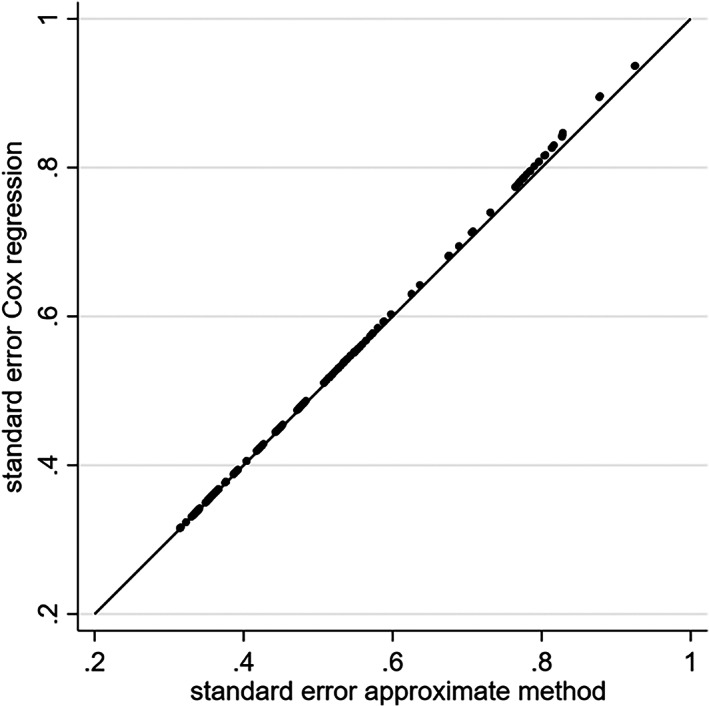
Comparison of SE of a treatment‐bone interaction from a Cox regression with those from our approximate method based on exponential regression, for the prostate cancer trial for increasing sample sizes from 50 participants (far right) to 502 participants (far left). The approximate method applied here sets $\exp\left(y_{\mathrm{ij}}\right)$ to be the mean follow‐up time in the participant's corresponding treatment and bone groups.

1. Option (i): set $w_{\mathrm{ij}}=e_{i}/n_{i}$ for every participant, where $e_{i}$ is the total number of observed outcome events in the trial and $n_{i}$ is the total sample size of the trial. This is equivalent to setting $w_{\mathrm{ij}}=1$ and replacing $n_{i}$ with $e_{i}$ in Equations (5) and (6). This approximation stems from Lemonte,^8^ who notes that $w_{\mathrm{ij}}=1$ when there are no censored observations; thus, to acknowledge that there are censored observations, our approach replaces the effective sample size from $n_{i}$ to $e_{i}$. This is a simple (pragmatic) approach, as the total number of outcome events and total sample size should be routinely available from a trial's publication (unlike the mean follow‐up time as in options (ii) and (iii) below). To examine this approximation, we replicated the previous simulation study using the prostate cancer trial data, but now compared the SE of the treatment‐age and treatment‐bone interactions from this approximate method (i.e., based on the exponential regression solutions of Equations (5) and (6), but using $w_{\mathrm{ij}}=1$ and changing $n_{i}$ to $e_{i}$) to those from fitting a Cox regression model. The results are shown in Figure 3 (i) and (ii), and the approximate method performs reasonably well, but is not perfect (differences between 10% and 16%), with greater differences to Cox regression in trials with a smaller sample size. Nevertheless, the approach is a reasonable approximation and practical to implement.
2. Option (ii): use $w_{\mathrm{ij}}=\exp\left(y_{\mathrm{ij}})\exp({\hat{\mu }}_{\mathrm{ij}}\right)$ and set $\exp\left(y_{\mathrm{ij}}\right)$ to be the mean follow‐up time in the participant's corresponding treatment group. We repeated the previous simulation study to compare SEs from a Cox regression model with those from this approximate method (i.e., based on an exponential regression and replacing $\exp\left(y_{\mathrm{ij}}\right)$ to be the mean follow‐up time in the participant's corresponding treatment group, with $\exp\left({\hat{\mu }}_{\mathrm{ij}}\right)$ derived from the fitted exponential regression model. Figure 3 (iii) and (iv) show the results are similar to option (i), in that the approximate method gives reasonably similar SEs to those from Cox regression, but not perfect. For example, for bone metastases (Figure 3 (iv)), with the SEs from the approximate method always smaller than those from the Cox model, between about 7% and 20%.
3. Option (iii) (for binary covariates): for each participant set $\exp\left(y_{\mathrm{ij}}\right)$ to be the mean follow‐up time in the subgroup defined by the participant's treatment and binary covariate classification. For example, in the prostate cancer trial data, this requires the mean follow‐up for each of four groups defined by treatment and bone group status. This method gives SEs in close agreement to those from the Cox regression (Figure 4), and highlights the improvement when using a more exact value of $\exp\left(y_{\mathrm{ij}}\right)$ for each participant. However, a downside is that the extra aggregate data (mean follow‐up for each of the four groups) is unlikely to be routinely reported.

## 3 CALCULATING THE POWER OF A POTENTIAL IPD META‐ANALYSIS PROJECT TO ESTIMATE A TREATMENT‐COVARIATE INTERACTION WITH A TIME‐TO‐EVENT OUTCOME

We now consider a how to undertake a power calculation for an IPD meta‐analysis project that aims to estimate a summary treatment‐covariate interaction estimate based on combining IPD from S parallel‐group randomised trials (e.g., by a previous systematic review). The premise is to do this *before* IPD collection, based on routinely reported aggregate data from the publications of trials already identified as relevant (e.g., from a previous systematic review) for potential inclusion in the IPD meta‐analysis project. Crucially, this means the power calculation is tailored to the actual known characteristics (e.g., total sample size and outcome events) of each trial whose IPD will be sought (or is even already promised). The power calculation involves five steps, which utilise the theory outlined in Section 2 for deriving the (unit) information matrix and variance matrix for a single trial.

The user needs to provide aggregate data for each trial (step 1), and the assumed interaction size (step 2), and then our Stata and R code implement the remaining three steps in under a minute. Stata code is provided in the Data S1, and R code is available at www.github.com/gscollins1973, for our applied example in Section 4.

### 3.1 Step 1: Extract aggregate data for each trial

For each trial potentially contributing their IPD for the IPD meta‐analysis project, we want to approximate the trial's information matrix ($I_{i})$ based on aggregate data. It is clear from Equation (3) that $I_{i}$ depends on the total participants, and the joint distribution of $x_{\mathrm{ij}}$, $z_{\mathrm{ij}}$ and $w_{\mathrm{ij}}$. Thus, the first step is to extract the following aggregate data from each trial:

- Total participants in the trial ($n_{i})$
- Total participants in the control group ($n_{Ci})$ and treatment group ($n_{Ti})$
- Number of outcome events in total ($e_{i})$
- Number of events in the control group ($e_{Ci})$ and treatment group ($e_{Ti})$*
- Mean follow‐up time in the control group ($f_{Ci})$ and treatment group ($f_{Ti})$*
- Characteristics that summarise the joint distribution of $x_{\mathrm{ij}}$ and $z_{\mathrm{ij}}$.

For a binary $z_{\mathrm{ij}}$, we require:

- Proportion of patients in the trial with $z_{\mathrm{ij}}$= 0 and $x_{\mathrm{ij}}$ = 0 $\left(\mathrm{Pr}\left(x=0,z=0\right)\right)$
- Proportion of patients in the trial with $z_{\mathrm{ij}}$ = 0 and $x_{\mathrm{ij}}$ = 1 $\left(\mathrm{Pr}\left(x=1,z=0\right)\right)$
- Proportion of patients in the trial with $z_{\mathrm{ij}}$ = 1 and $x_{\mathrm{ij}}$ = 0 $\left(\mathrm{Pr}\left(x=0,z=1\right)\right)$
- Proportion of patients in the trial with $z_{\mathrm{ij}}$ = 1 and $x_{\mathrm{ij}}$ = 1 $\left(\mathrm{Pr}\left(x=1,z=1\right)\right)$

For a continuous $z_{\mathrm{ij}}$, we typically assume a normal distribution and require:

- mean and SD of $z_{\mathrm{ij}}$ for $x_{\mathrm{ij}}$ = 0
- mean and SD of $z_{\mathrm{ij}}$ for $x_{\mathrm{ij}}$ = 1

‘*’ denotes not required if using option (i) in step 3—see below.

This set of aggregate data are usually available from trial publications, especially for commonly reported baseline covariates like sex and age (e.g., Table 1 of a trial publication usually summarises participant characteristics per group). Sometimes other information might be needed to derive the required aggregate data indirectly. For example, the mean follow‐up time can be derived from the total follow‐up time divided by the number of participants. Trial investigators can also be contacted to provide any missing information, and some could be derived from other results.

**TABLE 1:** Aggregate data from 10 randomised trials included in an IPD meta‐analysis project examining the effect of anti‐hypertensive treatment.

Trial	Total participants (events) control	Total participants (events) treatment	Mean follow‐up in years control	Mean follow‐up in years treatment	Age in years: mean (SD) control	Age in years: mean (SD) treatment	Male, % control	Male, % treatment
1	750 (13)	780 (9)	2.92	3.14	42.36 (5.34)	42.17 (5.39)	70.00	69.36
2	199 (28)	150 (27)	4.43	4.79	69.57 (5.39)	69.71 (5.18)	37.19	32.67
3	82 (29)	90 (32)	4.13	4.56	74.11 (8.69)	72.64 (7.99)	20.73	25.56
4	2371 (82)	2427 (81)	4.91	4.92	41.54 (5.48)	41.58 (5.53)	53.73	54.64
5	3445 (69)	3546 (73)	4.97	4.95	45.17 (5.86)	45.38 (6.00)	59.01	58.83
6	1337 (199)	1314 (178)	5.75	5.79	70.43 (2.72)	70.41 (2.74)	41.81	41.25
7	2371 (242)	2365 (213)	4.31	4.34	71.54 (6.68)	71.64 (6.72)	42.68	43.72
8	131 (7)	137 (4)	1.93	1.98	75.90 (3.95)	76.00 (3.75)	24.43	27.01
9	1139 (82)	1252 (61)	2.54	2.80	66.77 (5.67)	66.42 (5.34)	63.65	65.02
10	2297 (137)	2398 (123)	2.49	2.50	70.21 (6.67)	70.26 (6.73)	33.83	32.53

Abbreviation: IPD, individual participant data.

For a binary covariate, the joint distribution of $x_{\mathrm{ij}}$ and $z_{\mathrm{ij}}$ is defined exactly by the proportions $\mathrm{Pr}\left(x=0,z=0\right)$, $\mathrm{Pr}\left(x=0,z=1\right)$, $\mathrm{Pr}\left(x=1,z=0\right)$, and $\mathrm{Pr}\left(x=1,z=1\right)$. However, for a continuous covariate the underlying distribution also needs to be assumed; for simplicity, this will typically be assumed to be a normal distribution based on the reported mean and SD (perhaps after assuming a particular transformation), but it does not necessarily need to be (e.g., if a skewed distribution is more appropriate and can be approximated from other available summary statistics). If the distribution of $z_{\mathrm{ij}}$ is only summarised overall (i.e., not by $x_{\mathrm{ij}}$ = 1 and $x_{\mathrm{ij}}$ = 0 groups separately) then, as these are randomised trials, this distribution could be assumed the same for both treatment and control groups.

### 3.2 Step 2: Define the assumed true value of the treatment‐covariate interaction in each trial ($\lambda_{i})$

For the key parameter ($\lambda_{i})$, we suggest assuming a minimally important value, as identified via discussion with clinical experts within the IPD meta‐analysis project team. It is simplest to assume λ is common for all trials (see our consideration on between‐study heterogeneity in the Discussion).

### 3.3 Step 3: Estimate the variance of ${\hat{\lambda }}_{i}$ by simulating a large dataset that matches the aggregate data for each trial and deriving Fisher's unit information matrix

The next step is to approximate Fisher's unit information matrix and then the variance of ${\hat{\lambda }}_{i}$ for each trial separately, by applying the following process, which is implemented in our Stata and R code:

- Generate a large dataset of, say, 1 million participants each with a value of $x_{\mathrm{ij}}$ and $z_{\mathrm{ij}}$ sampled from distributions to reflect the extracted trial aggregate data from step 1, in terms of:
  - the proportion of participants in the treatment and control groups,
  - the proportion with an outcome event in each group,
  - the distribution of $z_{\mathrm{ij}}$ in each group (e.g., for a binary covariate, the correct $\mathrm{Pr}\left(x=0,z=0\right)$, $\mathrm{Pr}\left(x=0,z=1\right)$, $\mathrm{Pr}\left(x=1,z=0\right)$, and $\mathrm{Pr}\left(x=1,z=1\right)$; or for a continuous covariate, the correct mean and SD in each group)
- Generate $w_{\mathrm{ij}}$ for each participant in the dataset. Recall that $w_{\mathrm{ij}}=\exp\left(y_{\mathrm{ij}}+{\hat{\mu }}_{\mathrm{ij}}\right)=\exp\left(y_{\mathrm{ij}}\right)\exp\left({\hat{\mu }}_{\mathrm{ij}}\right),$ and we already discussed three options to approximate $w_{\mathrm{ij}}$ in Section 2.4. The first two options are the most practical:
  - Option (i): for each participant set $w_{\mathrm{ij}}=e_{i}/n_{i}$. This is the simplest option and does not even require assumptions about the parameter values of the exponential regression model for each trial.
  - Option (ii): for each participant, derive $w_{\mathrm{ij}}=\exp\left(y_{\mathrm{ij}}\right)\exp\left({\hat{\mu }}_{\mathrm{ij}}\right)$, after calculating $\exp\left(y_{\mathrm{ij}}\right)$ and $\exp\left({\hat{\mu }}_{\mathrm{ij}}\right)$ separately. First, set $\exp\left(y_{\mathrm{ij}}\right)$ for each participant as the mean follow‐up time in the participant's corresponding treatment group ($f_{Ti}$ or $f_{Ci}$). Then, derive $\exp\left({\hat{\mu }}_{\mathrm{ij}}\right)=\exp\left({\hat{\alpha }}_{i}+{\hat{\beta }}_{i}x_{\mathrm{ij}}+{\hat{\gamma }}_{i}z_{\mathrm{ij}}+{\hat{\lambda }}_{i}x_{\mathrm{ij}}z_{\mathrm{ij}}\right)$, which requires the user to specify the anticipated values of $\alpha_{i}$, $\beta_{i}$, $\gamma_{i}$, and $\lambda_{i}$. Without loss of generalisability, assume $z_{\mathrm{ij}}$ is centred by its mean. Then, ${\hat{\alpha }}_{i}$ becomes the log‐rate of the outcome event in the control group for a participant with the mean value of $z_{\mathrm{ij}}$, and can be derived from the extracted aggregate data using $e_{Ci}/\left(n_{Ci}f_{Ci}\right)$. The treatment effect (${\hat{\beta }}_{i}$) for a participant with the mean value of $z_{\mathrm{ij}}$ can be approximated by the rate ratio, as derived from the aggregate data using $\left(e_{Ti}/\left(n_{Ti}f_{Ti}\right)\right)/\left(e_{Ti}/\left(n_{\mathrm{Ti}}f_{Ti}\right)\right)$. In terms of the prognostic effect (${\hat{\gamma }}_{i})$ of the covariate, we suggest assuming this is zero for simplicity, or considering a range of possible of values (see examples later). The value of ${\hat{\lambda }}_{i}$ was defined in step 2.
- For each participant in the dataset, calculate the value of each element of B from Equation (5), and then calculate the mean value for each element. These mean values provide the corresponding values of each element of $I_{i}^{*}$ as defined in Equation (5).
- calculate the anticipated variance of the treatment‐covariate interaction for each trial, using Equation (6) of $\mathrm{var}\left({\hat{\lambda }}_{i}\right)=I_{i}^{*-1}\;\left(4,4\right)/n_{i}$.

### 3.4 Step 4: Estimate the variance of the summary treatment‐covariate interaction from the planned IPD meta‐analysis

Step 3 produces S estimates of $\mathrm{var}\left({\hat{\lambda }}_{i}\right)$, one for each trial, and we can now use these to derive the variance of the summary treatment‐covariate interaction estimate from an IPD meta‐analysis. We focus on using a two‐stage IPD approach. In the first stage, the treatment‐covariate interactions are estimated using the IPD in each trial separately; in the second stage, these interaction estimates are then pooled using a chosen meta‐analysis model.^2^ For example, a common‐effect model assumes the true interaction (λ) is assumed the same in all trials (i.e., $\lambda_{i}=\lambda$), such that,

(7) $${\hat{\lambda }}_{i}∼N\left(\lambda ,\mathrm{var}\left({\hat{\lambda }}_{i}\right)\right)$$

and the corresponding variance of the summary interaction estimate is:

(8) $$\mathrm{var}\left(\hat{\lambda }\right)=\frac{1}{\sum_{i=1}^{S}{\left(\mathrm{var}\left({\hat{\lambda }}_{i}\right)\right)}^{-1}}$$

Hence, the anticipated $\mathrm{var}\left(\hat{\lambda }\right)$ from a two‐stage meta‐analysis of interaction estimates can be obtained by simply plugging in the $\mathrm{var}\left({\hat{\lambda }}_{i}\right)$ derived following step 2 into Equation (8).

### 3.5 Step 5: Calculate the power of the planned IPD meta‐analysis

The final step is to calculate the power of the planned IPD meta‐analysis project to detect λ. Assuming a common interaction for all trials, and based on a Wald‐test and a 5% statistical significance level, the power is approximately:

(9) $$\begin{array}{rcl} \text{Power} & = & \text{Prob}\left(\frac{\hat{\lambda }}{\sqrt{\mathrm{var}\left(\hat{\lambda }\right)}}>1.96\right)\\ & & +\;\text{Prob}\left(\frac{\hat{\lambda }}{\sqrt{\mathrm{var}\left(\hat{\lambda }\right)}}<-1.96\right)\\ & & =\Phi \left(-1.96+\frac{\hat{\lambda }}{\sqrt{\mathrm{var}\left(\hat{\lambda }\right)}}\right)\;+\Phi \left(-1.96-\frac{\hat{\lambda }}{\sqrt{\mathrm{var}\left(\hat{\lambda }\right)}}\right) \end{array}$$

Here, $\Phi \left(x\right)$ is the probability of sampling a value < x from a standard normal distribution, $\mathrm{var}\left(\hat{\lambda }\right)$ is the variance of the summary interaction estimate (as obtained in step 2), and $\hat{\lambda }$ can be replaced with the assumed true λ (as defined in step 1). The power estimate is usually multiplied by 100 and reported as a %.

## 4 APPLIED EXAMPLE: IPD META‐ANALYSIS TO EXAMINE WHETHER SEX OR AGE INTERACTS WITH THE EFFECT OF ANTI‐HYPERTENSIVE TREATMENT

We now apply our proposed method to calculate the power of an IPD meta‐analysis to examine whether the effect of anti‐hypertensive treatment on mortality depends on either age or sex; that is, whether there is a treatment‐age or treatment‐sex interaction. Ten randomised trials of anti‐hypertensive treatment versus control were obtained, and the IPD meta‐analysis dataset has been analysed in various applied and methodology papers. Here, we focus on the power of this IPD meta‐analysis, but pretend that IPD are not yet available, and just use aggregate data about the 10 trials as previously reported (Table 1), and derive power using the four steps outlined in Section 3.

Stata code for this example is provided in Data S1, and R code is available at www.github.com/gscollins1973. These can easily be adapted for researchers in their own applications.

### 4.1 Step 1: Extract aggregate data

Using information from previous publications, we obtained aggregate data for the 10 trials as shown in Table 1, in terms of the number of participants, events and mean follow‐up per group, together with the mean and SD of age, and the percentage of males.

### 4.2 Step 2: Specify assumed value of $\lambda_{i}$

We assume $\lambda_{i}$ is common for all trials, and consider values for $\lambda_{i}$ of log(1.3) for sex (males compared to females) and log(1.3) for a 10‐year increase in age, assumed to be minimally important interactions to detect, for illustrative purposes.

### 4.3 Step 3: Estimate the variance of ${\hat{\lambda }}_{i}$ for each trial

Using our Stata or R code, which follows the approach outlined in Step 3 of Section 3, we used the aggregate data from step 1 to obtain the variance of ${\hat{\lambda }}_{i}$ for each study. Age was assumed normally distributed. Both option (i) and (ii) were used to calculate $w_{\mathrm{ij}}$. For option (ii), we assumed that there is no prognostic effect of the covariate ($\gamma_{i}=0)$. The estimated $\mathrm{var}\left({\hat{\lambda }}_{i}\right)$ for each trial is shown in Table 2, for each of sex and age, based on option (i) (i.e., assuming $w_{\mathrm{ij}}=e_{i}/n_{i}$). Results for option (ii) are very similar (see Table S1).

**TABLE 2:** Results of our power calculation for the anti‐hypertensive example, using the four‐step process described in Section 3 based on the aggregate data shown in Table 1 and applying option (i) to derive $w_{\mathrm{ij}}$ (i.e., $w_{\mathrm{ij}}=e_{i}/n_{i}\;).$

Study	Variance of each trial's interaction estimate ($\mathrm{var}\left({\hat{\lambda }}_{i}\right))$		Power (%) based on each trial separately		Weight (%) in the planned IPD meta‐analysis	
	Sex	Age	Sex	Age	Sex	Age
1	0.861	0.0063	5.92	6.25	1.17	1.14
2	0.329	0.0027	7.43	87.99	3.06	2.69
3	0.374	0.00095	7.13	13.65	2.69	7.58
4	0.099	0.00082	13.28	15.17	10.17	8.88
5	0.116	0.00080	12.00	15.25	8.64	8.95
6	0.044	0.0014	24.11	10.69	23.00	5.04
7	0.036	0.00020	28.34	46.60	28.05	36.67
8	1.910	0.0246	5.41	5.32	0.53	0.29
9	0.122	0.00093	11.66	13.88	8.23	7.78
10	0.069	0.00034	16.90	29.41	14.47	20.97

Planned IPD meta‐analysis	Variance of summary interaction estimate ($\mathrm{var}\left(\hat{\lambda }\right))$		Power (%) of planned IPD meta‐analysis	
	Sex	Age	Sex	Age
All 10 trials	0.010	0.000072	74.4%	87.2%

*Note*: For option (i), the mean follow‐up from in Table 1 is not required in the power calculation.

Abbreviation: IPD, individual participant data.

### 4.4 Step 4: Estimate the variance of the summary treatment‐covariate interaction from meta‐analysis

Based on a common‐effect meta‐analysis model, we applied Equation (8) to calculate the anticipated variance of the summary interaction estimate from the planned IPD meta‐analysis, which were 0.010 for sex and 0.000072 for age, for either options (i) or (ii).

### 4.5 Step 5: Calculate the power of the planned IPD meta‐analysis

Lastly, applying Equation (9), we calculated that the IPD meta‐analysis project has a power of 74.4% for sex and 87.2% for age. When using option (ii), the power was very similar (74.7% for sex and 87.3% for age—see Table S1). Had these power estimates been available before embarking on this IPD meta‐analysis project, it would have given strong reassurance to funders and the researchers that the project is worth the investment.

Some observations are worth noting. Firstly, the power is low for each trial separately, a consequence of each trial originally being powered on the overall treatment effect and not a treatment‐covariate interaction. This emphasises the importance of combining IPD from the multiple trials. Secondly, the anticipated contribution of each trial (as revealed by the percentage trial weights, Table 2) to the IPD meta‐analysis revealed which trials should be prioritised for their IPD. In particular, trials 6, 7 and 10 together contribute over 50% of the total weight, for either sex or age, and so are essential to obtain. The contribution of each trial is largest for those trials with more participants and events; however, a trial's contribution also depends on the variability of the covariate.^6^ For example, in the age power calculation, although trials 2 and 3 have a similar number of participants and events (Table 1), the percentage contribution (weight) in the IPD meta‐analysis of trial 3 is larger (7.6%) than for trial 2 (2.7%) because it has a larger SD of age (Table 2). Fourthly, although the power for sex is quite large and close to 80% based on the 10 trials, if additional IPD could be obtained from other anti‐hypertensive trials then this would still be worthwhile to improve the power further. Furthermore, if trials had longer follow‐up since the original trial publication, obtaining this updated IPD would also improve the power (due to additional events).

### 4.6 Sensitivity analysis

We repeated the power calculation for option (ii) rather assuming the prognostic effect of sex was $\gamma_{i}=\ln\left(1.25\right)$ and the prognostic effect of age was $\gamma_{i}=\ln\left(1.025\right)$. The power was similar, abeit slightly larger (sex = 75.2%, age = 88.1%), to the original analyses assuming the covariates had no prognostic effect.

Finally, we also considered option (iii) for the sex covariate, which aims to refine the value of $w_{\mathrm{ij}}$ for each participant by allowing for different $\exp\left(y_{\mathrm{ij}}\right)$ values for each of the four treatment and sex groupings. To implement this, whereas option (ii) set $\exp\left(y_{\mathrm{ij}}\right)$ to be the mean follow‐up in the participant's corresponding treatment group, for option (iii) we assumed a mean follow‐up time reduced by 0.25 years for males and increased by 0.25 for females compared to the overall mean follow‐up time in their respective treatment group. Implementing this, the power is estimated to be 74.4%, and so very similar to before. If we assume, more dramatically, that the mean follow‐up time would be reduced by 1 year for males and increased by 1 year for females, the power drops to 71.3%, but the general picture is consistent that the power is around 70%–75% regardless of whether options (i), (ii) or (iii) are used to derive $w_{\mathrm{ij}}$ for the power calculation.

## 5 DISCUSSION

In this paper we have proposed a new method to derive the power of a planned IPD meta‐analysis project aiming to estimate a treatment‐covariate interaction using randomised trials with a time‐to‐event outcome. This builds on our previous work focused on continuous and binary outcomes.^3^, ^5^ We outlined a five step approach that extracts aggregate data from trial publications; makes an assumption about the true (or minimally important) value of the treatment‐covariate interaction; derives Fisher's information matrix and an approximate estimate of the variance of each trial's interaction estimate; calculates the variance of the summary interaction estimate from a two‐stage IPD meta‐analysis; and calculates the power of the planned IPD meta‐analysis project. Our paper focused mainly on the development of the new method, and so further evaluation in other real examples and simulation studies would now be welcome.

We emphasise that our proposal is for use before IPD are obtained (i.e., during the design and planning stage), and assume that a meta‐analysis of interaction estimates is unavailable unless IPD are obtained. Without IPD, in order to perform a meta‐analysis of interaction estimates, the meta‐analysis researchers require ${\hat{\lambda }}_{i}$ and $\mathrm{var}\left({\hat{\lambda }}_{i}\right)$ to be available from trial publications. This is unlikely, as most trials focus on the overall treatment effect, and will not report treatment‐covariate interactions (especially those that are not significant). This motivates the collection of IPD for meta‐analysis, as it allows ${\hat{\lambda }}_{i}$ and $\mathrm{var}\left({\hat{\lambda }}_{i}\right)$ to be calculated directly, and our power calculation helps to inform whether the IPD collection is worth the investment for this purpose. IPD meta‐analysis projects may still be valuable even if the power is low, for example, to best summarise the uncertainty in existing evidence and to guide further research, but the power calculation helps provide more context for the decision to proceed or not.

We focused on a two‐stage approach to IPD meta‐analysis as, by pooling interaction estimates derived from solely within‐trial information (i.e., based at the participant‐level), this avoids trial‐level confounding and aggregation bias that may occur in meta‐regression based on across‐trial information,^11^, ^12^ or in one‐stage IPD meta‐analysis models that do not separate out within‐trial and across‐trial relationships. When specified correctly with the same assumptions (and estimation methods), one‐stage models and two‐stage models should agree closely unless most studies in the IPD meta‐analysis are small.^13^, ^14^ Therefore, the power calculation proposed should be applicable in most situations regardless of whether a one‐stage or two‐stage approach is ultimately used after IPD are obtained.

We also assumed a common‐effect meta‐analysis model in the second stage, which assumes the true interaction is the same in each study. This is a pragmatic approach, as otherwise accounting for heterogeneity would require assumptions about the magnitude of heterogeneity, and the variance of the meta‐analysis result would need to account for the uncertainty in the heterogeneity estimate in practice. This is unnecessarily complex for a power calculation onwards, in our opinion, but consideration of how to allow for heterogeneity is detailed elsewhere.^6^

A key issue for implementing the proposal is to obtain the necessary aggregate data for each group in each trial (Step 1 of our procedure). The number of participants and events should be available for each group. Information about covariate distributions may be more problematic. Standard covariates like age and sex should be summarised in each trial's table of baseline characteristics (often referred to as ‘Table 1’), but other covariates may not be. In this situation, trial investigators should be asked to supply the summary information needed for the covariates of interest. If they have promised their IPD (and thus are willing to collaborate on the IPD meta‐analysis project), this should not be an onerous task for them.

We provided various options for deriving, based on trial aggregate data, the variance of interaction estimates for each trial under the assumption of an exponential survival distribution. Options (i) and (ii) are the most pragmatic, especially option (i) which simply sets $w_{\mathrm{ij}}$ to be $e_{i}/n_{i}$. Either approach is an approximation (e.g., SEs were off by 10%–20% in the applied example of Figure 3), but it still provides a pragmatic starting point. Without IPD, knowing the actual distribution of $\exp\left(y_{\mathrm{ij}}\right)$ is hugely challenging, but if more refined values can be used, then they should (see Figure 4). Further research might consider using extracted information from published Kaplan–Meier curves (e.g., event and censoring times for each treatment group) for this purpose.

Further research should also evaluate the proposed method in situations with strong departures from the exponential survival distribution. Although we examined our method in real datasets, including where there was moderate deviation from a constant hazard (Figure 1), the method is likely to perform less well in situations where the exponential assumption (and thus a constant hazard) is unsuitable. In ‘A conversation with Sir David Cox’ by Reid,^15^ Cox states that he generally prefers specifying survival models parametrically as ‘various people have shown that the answers are very insensitive to the parametric formulation of the underlying distribution’. Whether this also applies to our method could be evaluated in simulation studies.

In summary, we hope our proposal (and associated Stata and R code) encourages readers to consider calculating the power of their planned IPD meta‐analysis projects to examine treatment‐covariate interactions with time‐to‐event outcomes. Power is an important aspect in deciding whether the IPD project is worth the investment, to be considered alongside other potential reasons for why IPD adds value compared to a traditional aggregate data meta‐analysis.^1^

## AUTHOR CONTRIBUTIONS

**Gary Stephen Collins:** Formal analysis; funding acquisition; methodology; software; writing – review and editing. **Miriam Hattle:** Methodology; writing – review and editing. **Rebecca Whittle:** Methodology; software; writing – review and editing. **Joie Ensor:** Formal analysis; funding acquisition; methodology; software; writing – review and editing. **Richard Riley:** Conceptualization; data curation; formal analysis; funding acquisition; methodology; software; writing – original draft; writing – review and editing.

## FUNDING INFORMATION

Richard D. Riley, Miriam Hattle, Gary S. Collins and Joie Ensor were supported by funding from the MRC Better Methods Better Research panel (grant reference: MR/V038168/1). Gary S. Collins was supported by Cancer Research UK (programme grant: C49297/A27294). Richard D. Riley and Joie Ensor are supported by the NIHR Birmingham Biomedical Research Centre (BRC) at the University Hospitals Birmingham NHS Foundation Trust and the University of Birmingham. The views expressed are those of the authors and not necessarily those of the NHS, the NIHR or the Department of Health and Social Care.

## CONFLICT OF INTEREST STATEMENT

Richard Riley is the lead Editor on the book ‘Individual Participant Data Meta‐Analysis: A Handbook for Healthcare Research’ for which he receives royalties.

## Supporting information


**DATA S1.** Stata and R code.

**TABLE S1.** Results of our power calculation for the anti‐hypertensive example, using the four‐step process described in Section 3 based on the aggregate data shown in Table 1* and applying option (ii) to derive $w_{\mathrm{ij}}$.

## Data Availability

The IPD for the simulation study are from a randomised trial evaluating the use of oestrogen for the treatment of Stage 3 or 4 prostate cancer patients, and this is available from http://hbiostat.org/data courtesy of the Vanderbilt University Department of Biostatistics. Aggregate data for the applied IPD meta‐analysis example is provided in the supplementary material, along with Stata code. R code is available at www.github.com/gscollins1973.

## References

[1] Riley RD , Tierney JF , Stewart LA , eds. Individual Participant Data Meta‐Analysis: A Handbook for Healthcare Research. Wiley; 2021.

[2] Simmonds MC , Higgins JP . Covariate heterogeneity in meta‐analysis: criteria for deciding between meta‐regression and individual patient data. Stat Med. 2007;26(15):2982‐2999. 17195960 10.1002/sim.2768

[3] Riley RD , Hattle M , Collins GS , Whittle R , Ensor J . Calculating the power to examine treatment‐covariate interactions when planning an individual participant data meta‐analysis of randomized trials with a binary outcome. Stat Med. 2022;41:4822‐4837. 35932153 10.1002/sim.9538 PMC9805219

[4] Kovalchik SA , Cumberland WG . Using aggregate data to estimate the standard error of a treatment‐covariate interaction in an individual patient data meta‐analysis. Biom J. 2012;54(3):370‐384. 22685003 10.1002/bimj.201100167

[5] Riley RD , Debray TPA , Fisher D , et al. Individual participant data meta‐analysis to examine interactions between treatment effect and participant‐level covariates: statistical recommendations for conduct and planning. Stat Med. 2020;39(15):2115‐2137. 32350891 10.1002/sim.8516 PMC7401032

[6] Riley RD , Ensor J . Power calculations for planning an IPD meta‐analysis. In: Riley RD , Tierney J , Stewart LA , eds. Individual Participant Data Meta‐Analysis: A Handbook for Healthcare Research. Wiley; 2021.

[7] Kalbfleisch JD , Prentice RL . The Statistical Analysis of Failure Time Data. 2nd ed. J. Wiley; 2002 xiii, 439 p.

[8] Lemonte AJ . Covariance matrix of maximum likelihood estimators in censored exponential regression models. Commun Stat Theory Methods. 2022;51(6):1765‐1777.

[9] Byar DP , Green SB . The choice of treatment for cancer patients based on covariate information. Bull Cancer. 1980;67(4):477‐490. 7013866

[10] Andrews DF . In: Herzberg AM , ed. Data: A Collection of Problems from Many Fields for the Student and Research Worker. Springer‐Verlag; 1985.

[11] Fisher DJ , Copas AJ , Tierney JF , Parmar MK . A critical review of methods for the assessment of patient‐level interactions in individual participant data meta‐analysis of randomized trials, and guidance for practitioners. J Clin Epidemiol. 2011;64(9):949‐967. 21411280 10.1016/j.jclinepi.2010.11.016

[12] Thompson SG , Kaptoge S , White I , Wood A , Perry P , Danesh J . Statistical methods for the time‐to‐event analysis of individual participant data from multiple epidemiological studies. Int J Epidemiol. 2010;39(5):1345‐1359. 20439481 10.1093/ije/dyq063 PMC2972437

[13] Burke DL , Ensor J , Riley RD . Meta‐analysis using individual participant data: one‐stage and two‐stage approaches, and why they may differ. Stat Med. 2017;36(5):855‐875. 27747915 10.1002/sim.7141 PMC5297998

[14] Riley RD , Burke DL , Morris TP . One‐stage versus two‐stage approach to IPD meta‐analysis: differences and recommendations. In: Riley RD , Tierney JF , Stewart LA , eds. Individual Participant Data Meta‐Analysis: A Handbook for Healthcare Research. Wiley; 2021.

[15] Reid N . A conversation with Sir David Cox. Stat Sci. 1994;9(3):439‐455.

