# Supplementary material for: Calculating the power of a planned individual participant data meta‐analysis of randomised trials to examine a treatment‐covariate interaction with a time‐to‐event outcome
Source: Res Synth Methods. 2023 Jun 29;14(5):718–30. doi: 10.1002/jrsm.1650 (PMC10947306; doi:10.1002/jrsm.1650)
Supplement: Supplementary file 2 — TABLE S1. Results of our power calculation for the anti‐hypertensive example, using the four‐step process described in Section 3 based on the aggregate data shown in Table 1* and applying option (ii) to derive wij. [file JRSM-14-718-s002.docx]

## Supplementary Material

[Note to production: the Supplementary table should be set as part of the main paper]

**Stata code** **and aggregate dataset** – see online Supporting Information files.

**Supplementary Table:** Results of our power calculation for the anti-hypertensive example, using the four-step process described in Section 3 based on the aggregate data shown in Table 1* and applying option (ii) to derive $w_{ij}$

|  | **Variance of each trial’s interaction estimate (**${\mathbf{var}\boldsymbol{(}\hat{\boldsymbol{\lambda}}}_{\boldsymbol{i}}\boldsymbol{))}$ | | **Power (%) based on each trial separately** | | **Weight (%) in the planned IPD meta-analysis** | |
| --- | --- | --- | --- | --- | --- | --- |
| **Study** | **Sex** | **Age** | **Sex** | **Age** | **Sex** | **Age** |
| 1 | 0.950 | 0.00647 | 5.83 | 6.23 | 1.05 | 1.11 |
| 2 | 0.309 | 0.00260 | 7.59 | 8.09 | 3.24 | 2.76 |
| 3 | 0.354 | 0.00095 | 7.25 | 13.66 | 2.82 | 7.58 |
| 4 | 0.100 | 0.00080 | 13.14 | 15.23 | 9.95 | 8.91 |
| 5 | 0.120 | 0.00080 | 11.81 | 15.32 | 8.36 | 8.99 |
| 6 | 0.043 | 0.00143 | 24.42 | 10.68 | 23.26 | 5.02 |
| 7 | 0.035 | 0.00019 | 28.57 | 46.80 | 28.18 | 36.77 |
| 8 | 1.902 | 0.02683 | 5.42 | 5.29 | 0.53 | 0.27 |
| 9 | 0.131 | 0.00095 | 11.19 | 13.64 | 7.62 | 7.55 |
| 10 | 0.067 | 0.00034 | 17.40 | 29.56 | 14.99 | 21.04 |
|  | **Variance of summary interaction estimate (**$\mathbf{var}\left( \hat{\boldsymbol{\lambda}} \right)\boldsymbol{)}$ | | **Power (%) of planned IPD meta-analysis** | |  |  |
| **Planned IPD meta-analysis** | **Sex** | **Age** | **Sex** | **Age** |  |  |
| All 10 trials | 0.010 | 0.000072 | **74.7%** | **87.3%** |  |  |

* Assuming $\alpha$ is the observed log-odds of the outcome event in the control group, $\beta$ is the observed overall treatment effect (log odds ratio), there is no prognostic effect of the covariate ($\gamma=0)$ and $\lambda$ is log(1.3) for sex (males compared to females) and log(1.3) for a 10-year increase in age.
